# Severe Hyperhidrosis Secondary to Bupropion Use and Treatment. A case report.

**DOI:** 10.1192/j.eurpsy.2024.662

**Published:** 2024-08-27

**Authors:** A. Oliva Lozano, J. Garde Gonzalez, M. A. Morillas Romerosa, P. Herrero Ortega

**Affiliations:** ^1^Psychiatry, Hospital La Paz, Madrid, Spain

## Abstract

**Introduction:**

Hyperhidrosis, the excessive and uncontrollable sweating, is a well-documented side effect of various medications. Among these, bupropion, a commonly prescribed antidepressant and smoking cessation aid, has been associated with the development of severe hyperhidrosis in a subset of patients. This clinical report aims to shed light on a compelling case of severe hyperhidrosis induced by bupropion use and the subsequent treatment strategies employed

The patient under discussion is a 42-year-old female with a history of recurrent major depressive disorder and a previous favorable response to selective serotonin reuptake inhibitors (SSRIs). Due to side effect concerns and a desire to quit smoking, she was transitioned to bupropion, a norepinephrine-dopamine reuptake inhibitor (NDRI), at a standard therapeutic dose of 150 mg daily.

Approximately four weeks after initiating bupropion therapy, the patient began experiencing debilitating symptoms of excessive sweating, particularly affecting her palms, soles, and axillae. The profuse sweating episodes occurred throughout the day and night, significantly impairing her quality of life, social interactions, and occupational functioning. No previous history of hyperhidrosis was reported, and physical examinations revealed no underlying medical conditions or dermatological issues.

**Objectives:**

To aknowledge the importance of recognizing and addressing medication-induced side effects within the realm of psychiatry and an early implementation of patient-centered treatment.

**Methods:**

Clinical case report and a brief literature review.

**Results:**

The treatment of hyperhidrosis secondary to bupropion use presents a challenging clinical scenario that requires a delicate balance between managing the distressing side effect and ensuring the continued efficacy of psychiatric therapy. Given the rarity of severe hyperhidrosis as a side effect of bupropion, there is a limited body of evidence guiding treatment strategies. Gradual withdrawal in the dose of bupropion was initiated, with careful monitoring of depressive symptoms to prevent relapse, switching to Duloxetine 90mg daily, with adecuate efectiveness.In this particular case, the combination of medication adjustment and psychological support led to a significant reduction in hyperhidrosis symptoms. The patient reported improved social interactions, enhanced self-esteem, and restored occupational functioning. Importantly, her depressive symptoms remained well-managed, underscoring the success of the treatment strategy.

**Conclusions:**

This clinical report highlights the importance of a patient-centered approach when addressing rare medication-induced side effects within the field of psychiatry. Bupropion withdrawal and regular follow-up showed effective in the treatment of the simptoms. Future research may provide additional insights and treatment options for cases like this, further enhancing patient care and outcomes.

**Disclosure of Interest:**

None Declared

